# Interactions between taste and oral microbiome: mechanisms and implications for oral and systemic diseases

**DOI:** 10.1080/20002297.2026.2699527

**Published:** 2026-07-11

**Authors:** Mengpei Xi, Shaoying Li, Yumu Tang, Jianhui Zhu, Shuli Deng

**Affiliations:** a Stomatology Hospital, School of Stomatology, Zhejiang University School of Medicine, Zhejiang Provincial Clinical Research Center for Oral Diseases, Zhejiang Key Laboratory of Oral Biomedical, Hangzhou, China; b Department of Stomatology, Hainan Provincial Women and Children's Medical Center, Haikou, China; c Department of Endodontics, Suzhou Stomatological Hospital, Suzhou, China

**Keywords:** Oral microbiome, taste, taste receptor, systemic disease, oral disease

## Abstract

**Background:**

The oral microbiome is a complex microbial ecosystem that contributes to oral and systemic health. Emerging evidence suggests a bidirectional interaction between oral microbiota and taste, although its underlying mechanisms and disease implications remain incompletely understood.

**Objective:**

This review aims to summarize current knowledge regarding the interactions between the oral microbiome and taste, elucidate the potential mechanisms involved, and discuss their relevance to human diseases.

**Design:**

Recent advances in clinical and experimental studies were reviewed, focusing on microbial metabolism, immunoinflammatory regulation, taste receptor modulation, and microbial-host interactions.

**Results:**

Oral microorganisms may influence taste perception through metabolite production, inflammatory pathways, alteration of taste receptor expression, and other mechanisms such as physical barriers. Conversely, taste perception and taste receptors can regulate microbial colonization by shaping dietary behaviors and local immune responses. These interactions may contribute to the development and progression of oral diseases, extraoral inflammatory diseases, cardiometabolic disorders, cancer, and neurodegenerative conditions.

**Conclusions:**

The taste-oral microbiome axis represents an emerging regulatory network linking microbial ecology, sensory function, and disease pathogenesis. Further longitudinal and mechanistic studies are required to clarify causal relationships and explore microbiome-targeted therapeutic strategies.

## Introduction

The oral microbiome, the second-largest microbial community in humans after the gut, consists of a diverse range of microorganisms residing in the oral cavity. Previous research has demonstrated that oral microbiome and their interactions with the host are key influencing factors of both oral and general health [1,2]. They not only mediate local inflammatory pathologies in the oral cavity but also exert effects on distant sites, thereby affecting the development and progression of various systemic diseases [3,4]. Within this shared oral environment, the taste system operates as a critical physiological interface. It primarily functions to sense nutrients and noxious substances, and further regulates metabolism and immune responses through distinct mechanisms [5]. Accumulating evidence indicates a complex bidirectional interaction between the oral microbiota and taste perception [6–9]. Specifically, oral microorganisms can modulate taste receptor function and sensitivity, while shifts in taste perception may, in turn, influence microbial composition through altered dietary intake [10,11]. This intricate interaction network is increasingly recognized to be implicated in the onset, treatment and prognosis of both oral and systemic diseases [12].

This article summarizes recent advances in the study of the interactions between oral microbiota and the gustatory system, their underlying mechanisms and impact on health. The purpose of this review is to gain a better understanding of the pivotal roles of taste-oral microbiome interactions in health and disease, thereby providing novel perspectives for further investigations on therapeutic strategies by regulating the microbiota-taste axis.

### The oral microbiome

The human oral cavity contains a complex ecosystem that supports a diverse community of microorganisms, collectively referred to as the oral microbiome. Oral microbiome represents one of the most intricate microbial communities within the human body, consisting of viruses, fungi, protozoa, archaea and bacteria [3]. Among these, bacteria are the most abundant and most extensively studied microorganisms. According to the Human Oral Microbiome Database, there are over 700 species of oral bacteria, belonging to various genera distributed among seven major phyla: Actinomycetota, Bacteroidota, Bacillota, Fusobacteriota, Pseudomonadota, Saccharibacteria and Spirochaetota [13,14]. The dominant oral bacterial species are generally conserved across individuals, while bacterial diversity arises from differences in taxonomic abundance, strain-level variation and the presence of rare lineages [14]. The oral cavity is an open and dynamic system, which is continually exposed to food, beverages, air, exogenous microorganisms and human contact, making it a great challenge to accurately determine the precise composition of the oral microbiome.

In addition to microbial composition, the spatial and structural organization, or the so-called biogeography of microbial communities has garnered increasing scholarly attention. The oral microbiota establishes distinct microenvironments across various oral niches, such as the tooth surfaces, gingiva, tongue dorsum, buccal mucosa and saliva, maintaining local ecological balance through microbial interactions. As reviewed by Sedghi et al., most oral microbes exhibit site specialization, determined by their ability to adhere, establish, proliferate and replicate in specific locations [11]. For instance, among *Streptococcus* species, *S. parasanguinis* and *S. salivarius* are specialized for colonization on the tongue dorsal surfaces, whereas *S. sanguinis* and *S. gordonii* reside in dental plaque communities [15]. A recent review on biogeography of the oral microbiome concluded that, since microbes from all oral sites are shed into saliva, which is distributed throughout the oral cavity, most oral microorganisms can be detected at any oral site. Nevertheless, their relative abundance is significantly higher in locations considered as their true ecological niches [14]. The microbiotas of dental plaque, the tongue dorsum and the keratinized gingiva are thought to be the most distinctive from one another [15].

The oral and intestinal microbiota inhabit distinct regions of the digestive system and engage in complex interactions via hematogenous and enteral pathways [16]. Numerous studies have identified the colonization of oral bacteria in the gut. Given the anatomical continuity and the influence of chemical mediators such as saliva and dietary components, oral-resident bacteria may colonize the intestinal tract ectopically through the gastrointestinal pathway [17]. Additionally, growing evidence indicates that oral bacteria can translocate to the gastrointestinal tract via hematogenous routes [17,18]. For instance, research has demonstrated that the hematogenous route may be preferred by oral fusobacteria to reach colon tumors [19]. The oral and intestinal microbiota are intricately interconnected, collectively forming the ‘oral-gut microbiome axis’, which plays a crucial role in the pathogenesis and progression of diseases such as inflammatory bowel disease, obesity, heart failure and cancer [20–23].

### Taste system

Taste is the sensory system that enables organisms to identify nutrients and avoid toxins [24,25], significantly shaping their dietary choices. Taste buds located in the oral cavity can detect taste substances and produce taste signals which are transmitted to the brain through taste nerves [26]. Mammals can identify five basic tastes: sweet, salty, sour, bitter and umami, with accumulating evidence for fat as additional taste qualities [27,28].

Taste signals first arise via taste receptor cells (TRCs) in taste buds. These onion-like structures mainly reside within taste papillae (circumvallate, foliate and fungiform papillae) of the tongue, with a minority located on the palate, epiglottis, pharynx and larynx [29]. Each taste bud comprises roughly 100 cells [30], which are categorized into four types based on their ultrastructural features, gene expression profiles, and functional roles [26,31]. Type I cells act as glia-like cells, clearing extracellular neurotransmitters to maintain microenvironment homeostasis [32]. Type II cells express G protein-coupled receptors (GPCRs) that detect sweet, umami and bitter tastes. Type III cells primarily sense sour taste and form synapses with afferent fibers, while type IV cells function as progenitors to support taste cell renewal [5]. Taste signals initiated at the taste buds are conveyed via the facial, glossopharyngeal and vagus nerves to the nucleus solitarius (NTS), subsequently relaying through the thalamus and limbic system before reaching the insular taste cortex [33]. Notably, taste buds are capable of self-renewal through highly proliferating organ-specific stem cells. In mice, leucine-rich repeat-containing G protein-coupled receptor 5 (Lgr5) marks progenitor cells in the posterior part of the tongue while Lgr6 marks progenitor cells in both anterior and posterior tongue. Extensive studies on taste bud renewal in mice have shown that the Wnt/β-Catenin, SHH and FGF are primary signaling pathways regulating taste bud renewal [26].

Taste receptors mediate taste signal transmission by converting the chemical signals of tastants into electrical signals [34]. Sweet, umami and bitter tastes are detected by GPCRs and are transduce through a shared intracellular signaling pathway. Specifically, the sweet taste is recognized by the TAS1R2/TAS1R3 heterodimer, umami by the TAS1R1/TAS1R3 heterodimer and bitter by the TAS2Rs family [32]. In contrast, sour and salty tastes are detected through ion channels, with sour primarily sensed by the proton channel otopetrin 1 (OTOP1), and salty taste predominantly mediated by the epithelial sodium channel (ENaC) [5,26]. In addition, free fatty acids are considered stimuli for fat taste, with cluster of differentiation 36 (CD36) and G protein-coupled receptor 120 (GPR120) being identified as key mediators for this perception [35]. Notably, taste receptors are also distributed in extraoral tissues such as the gastrointestinal tract, bladder, brain, respiratory tract, heart, paranasal sinuses, thyroid gland and testicles [36]. Extraoral taste receptors have been found to be involved in the occurrence and development of various diseases. For instance, gustatory tanycytes are defined as a specialized subpopulation of ependymal cells in the hypothalamus that express bitter taste receptors in the brain's ventricular walls. These cells are capable of monitoring physiological states and regulating glucose homeostasis [37]. Activation of intestinal bitter taste receptor Tas2r108 remodels enteroendocrine hormone release and bile acid metabolism, thereby ameliorating metabolic syndrome features [38].

## Oral microbiota-taste interactions and the underlying mechanisms

Numerous studies have established a correlation between interindividual variations in oral microbiota and differences in taste perception (Table 1). Microbial presence or abundance is correlated with either enhanced or diminished taste sensitivity, and specific taste phenotypes are, in turn, linked to distinct microbial profiles. For instance, in a cross-sectional clinical study of acutely hospitalized elderly patients, Solemdal et al. reported that rapid proliferation of *Lactobacillus* in the oral cavity was associated with a measurable reduction in sour taste perception [9], illustrating a potential link between an overgrowth of a specific microbial group and the impairment of taste perception.

**Table 1.** Associations between oral microbes and taste perception.

Oral microbes	Associations with taste perception	Reference
Lactobacillus	Sour taste perception is reduced among acutely hospitalized elderly with rapid growth of Lactobacillus.	Solemdal et al. [9]
*Candida albicans*	The presence of *C. albicans* in the oral cavity is one of the causes of taste disorders.	Sakashita et al. [39]
Actinobacteria, Firmicutes, Bacteroidetes	The proportions of Actinobacteria and Firmicutes in saliva were negatively associated with taste sensitivity scores, and the proportion of Bacteroidetes in tongue film was positively associated with sweet, salty and bitter tastes.	Feng et al. [6]
Clostridiales, Bacteroidales	Specific taxa, mainly attributed to Clostridiales and Bacteroidales order, were inversely correlated with salt and sour thresholds.	Cattaneo et al. [10]
*Actinomyces*, *Oribacterium*, *Solobacterium*, *Catonella*, *Campylobacter*	PROP non-tasters show significant higher detection thresholds of sweet, sour, salty and bitter tastes than supertasters and five bacterial genera are significantly overrepresented in supertasters.	Cattaneo et al. [40]
*Streptococcus mutans*	Children with an elevated sucrose taste threshold were more likely to develop *S. mutans* presence.	Jurczak et al. [41]
*Streptococcus parasanguinis*, *Streptococcus gordonii*	The abundance of *Streptococcus* species, especially *S. gordonii* and *S. parasanguinis*, is correlated with a reduced taste sensitivity.	Licandro et al. [8]

**Figure 1.**
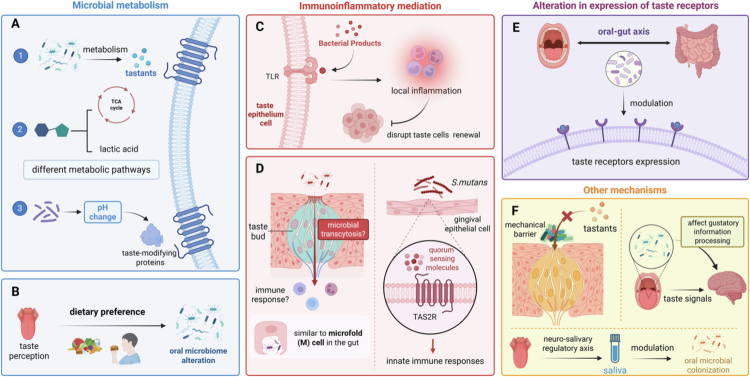
Mechanisms underlying taste-oral microbiome interactions. (A) Oral microorganisms can influence the gustatory system through microbial metabolism, including the direct metabolism of taste substances (which alters their concentration), the utilization of distinct metabolic pathways and modulation of the oral microenvironment's pH, ultimately affecting taste perception. (B) Taste perception plays a role in shaping oral microbial communities by influencing dietary preferences. (C) Certain microbial components interact with receptors such as TLRs in taste epithelium, thereby interfering with the renewal of taste cells through inflammatory pathways. (D) Taste cells are involved in immune surveillance like microfold cells in the intestine, and may adjust responses to microbial signaling and infection through microbial transcytosis. In gingival epithelial cells, bitter taste receptors are capable of detecting quorum sensing molecules (MSQ) from *Streptococcus mutans* and mediating innate immune responses. (E) The oral microbiome is in constant communication with gut microorganisms, which may influence taste perception by altering taste receptor expression. (F) The tongue film formed by microbial communities may act as a mechanical barrier. Certain microbiota and their metabolites are capable of crossing the blood–brain barrier, potentially affecting the processing of taste information in the central nervous system. Additionally, the neuro-salivary regulatory axis may modulate oral microbial colonization by altering the flow rate and compositional profile of saliva.

The interaction between the oral microbiota and the taste system is a bidirectional and dynamic process, and researchers have explored the potential mechanisms involved. Oral microbiota influence taste perception mainly through microbial metabolism, interaction with the host immune system, altering receptor expression and physical obstruction formation. Conversely, changes in taste perception and taste receptors may regulate the colonization and adhesion of oral microbiota by affecting dietary behavior and local immune microenvironment (Figure 1). The following section will elaborate on the specific mechanisms underlying these bidirectional interactions.

### Microbial metabolism

The metabolic activities of oral microorganisms can directly alter the concentration of tastants, thus influencing taste perception. Specifically, glutamate content is noted as a key contributor to the umami taste and can be produced by some oral bacteria [42]. *Porphyromonas gingivalis* and *Fusobacterium* species, possess enzymes to metabolize glutamate through diverse pathways, including deamination and decarboxylation reactions, thereby altering its basal concentration and consequently contributing to variations in umami perception [43]. Additionally, certain bacterial species, such as those within the Actinobacteria phylum, produce phenolic secondary metabolites that serve as precursors for bitter acids, significantly enhancing the perception of bitterness in foods and ultimately influencing sensory adaptation to bitter taste [40]. Research has also shown that the production of oral bacterial-derived short chain fatty acids (SCFAs) such as propionate contributes to oral microbiota's influence on taste perception [44]. For example, the concentrations of SCFAs correlate with bacterial load [45] and can affect the perception threshold of non-esterified fatty acids [42].

Variations in the metabolic pathways of oral microorganisms also contribute to differences in taste perception. Gander et al. focused on intraoral microbial metabolism and demonstrated that individuals with high sensitivity to sucrose exhibit increased citric acid cycle activity, whereas those with diminished sensitivity to sucrose maintain a more efficient conversion of pyruvate to lactic acid. This suggests that individual sensitivity to sucrose sweetness is linked to variations in the catabolic processing of sucrose within the oral cavity [46]. A subgroup analysis conducted on obese linoleic acid tasters (OT) and obese linoleic acid non-tasters (ONT) revealed that specific bacterial metabolic pathways, such as phosphotransferase and simple sugar transport systems, were more prevalent in ONT [47].

Oral microbiome may affect taste perception by other indirect metabolic mechanisms. For instance, the oral microbiome, particularly acid-producing bacteria, can alter oral environment by changing oral pH. This change in pH environment has been shown to correlate with sweet taste sensitivity [48,49]. Among proteins used as sweeteners, neoculin and miraculin are taste-modifying proteins that exhibit sweetness in a pH-dependent manner [50]. Additionally, SCFAs have also been demonstrated to upregulate the expression and activity of umami taste receptors in enteroendocrine cells via a Gα_i/o_ signaling pathway, potentially increasing the gut's sensitivity to dietary molecules [51].

Conversely, individual differences in taste perception may also influence the oral microbiota through the mediation of dietary choices. A population-based observational study has shown that individuals with high sugar intake exhibit enrichment of acid-tolerant and cariogenic bacteria such as *Streptococcus mutans* in their oral microbiome, along with heightened activity in sugar metabolic pathways [52]. Notably, such microbial changes are associated with specific variants of taste-related genes *TAS1R1* and *GNAT3*, suggesting that taste perception may indirectly modulate the composition of oral microorganisms by shaping dietary preferences. A preference for specific taste such as sweet or umami can increase the intake of corresponding nutrients (e.g. monosaccharides and amino acids) [53,54], which may provide a competitive advantage for specific oral microbes that utilize these nutrients such as sugar-metabolizing streptococci, thereby creating a favorable ecological niche and, to some extent, altering the structure of the oral microbial community.

### Immunoinflammatory mediation

Oral microbiota and taste epithelium interact through inflammatory mechanisms. The oral microbiome can induce both local and systemic immune responses, which may indirectly impact the function of taste receptors and taste perception. Research indicates that the circumvallate papillae (CVP) microbiota in linoleic acid non-tasters exhibits a higher capacity for inducing local inflammation compared to those in linoleic acid tasters [47]. Certain microorganisms are capable of mediating the secretion of inflammatory factors by host cells and inducing inflammation [12]. Studies have identified the expression of Toll-like receptors (TLRs), type I and II interferon (IFN) receptors and their downstream signaling components such as STAT1 and STAT2 in taste epithelium, with notably higher levels of these inflammatory receptors including TLRs compared to non-gustatory epithelial cells [55]. Lipopolysaccharides (LPS), as the cell wall component of Gram-negative bacteria, may influence the expression or function of taste receptors through inflammatory pathways. Acute intraperitoneal injection of LPS can induce the production of proinflammatory cytokines by taste buds, thereby inhibiting the proliferation of taste progenitor cells and disrupting the renewal of taste cells [56]. It is plausible that Gram-negative bacteria may directly or indirectly activate taste epithelial cells, thereby stimulating the production of inflammatory cytokines, which could contribute to the development of taste disorders associated with infections. Additionally, it has been found that TLR4 knockout mice exhibited reduced preferences for sweet, fat and umami tastes, along with decreased expression of key taste molecules such as CD36, PLC2β and TRPM5 in the tongue epithelium, suggesting certain inflammatory pathways play an important role in taste perception [57].

Conversely, taste cells and taste receptors may also exert influence on oral microbiota through host immune responses. Recent studies have demonstrated that taste cells are involved in immune surveillance like counterparts in the intestine, and may adjust responses to microbial signaling and infection [58,59]. Qin et al. discovered that type II taste cells express multiple microfold (M) cell marker genes, including *Spib*, a RANKL-regulated transcription factor essential for M cell development and regeneration [59]. Similar to M cells, taste cells from wild-type mice, but not from *Spib* knockout (*Spib* ^*KO*^) mice, exhibit the characteristics of microbial transcytosis. The study also observed that *Spib* ^*KO*^ mice show increased attraction to sweet and umami tastants, although the underlying mechanism remains unclear. In addition, taste receptors exhibit varying degrees of responsiveness to microbes and are implicated in host immune regulation. Using taste-transduction-deficient mice and ligature-induced periodontitis models, Zheng et al. demonstrated that gingival solitary chemosensory cells (gSCCs) are likely to respond to bacterial signals via Tas2rs and downstream taste signaling components, thereby triggering host innate immune responses [60]. For instance, bitter taste receptor TAS2R14 is capable of detecting quorum sensing molecules (MSQ) from cariogenic *Streptococcus mutans* and mediating innate immune responses in gingival epithelial cells [61,62].

### Alteration in expression of taste receptors

Studies have identified that gut microorganisms may influence taste perception by altering taste receptor expression, and the oral microbiome, which is in constant communication with gut microorganisms, may be a potential participant in this process. One study found that germ-free mice exhibit an increased preference for and caloric intake from fats, which is associated with an increase in oral receptors for fats and a broad and significant decrease in the expression of intestinal satiety peptides and fatty-acid receptors, compared to mice with normal microbiome [63]. Fecal transplantation from obese mice fed a high-fat diet reverses the reduction in fat mass and increase in lean body mass induced by a low-fat diet. This process also results in alterations in intestinal *Tas2r138* and *Tas2r116* mRNA levels, supporting the hypothesis that these receptors are modulated by diet-induced changes in bacterial composition [64]. Swartz et al. demonstrated an upregulation of intestinal Tas1r3 and sodium glucose luminal transporter-1 (SGLT-1) expression, along with increased sucrose intake, in germ-free mice compared to conventional mice [65]. However, the mRNA expression of tongue taste receptors (Tas1r2, Tas1r3) was similar between the two groups.

### Other mechanisms

Beyond these mechanisms discussed above, other mechanisms may also contribute to the taste-oral microbiome interactions. The tongue film formed by microbial communities on the dorsal surface of the tongue, predominantly consisting of oral bacteria, may act as a mechanical barrier, potentially interfering with taste receptor accessibility to tastants and downstream signaling [7,66]. The pseudomembranes resulting from the excessive growth of fungi such as *Candida albicans* can also create mechanical barriers, thereby influencing taste perception [33].

Furthermore, the oral microbiota may influence taste perception through indirect effects on distal organs. Notably, patients with neurodegenerative diseases, including Alzheimer's disease and Parkinson's disease, have frequently been reported to exhibit both dysregulated oral microbiota composition and impaired gustatory function [67,68], suggesting a potential clinical association between oral microbial imbalance and sensory dysfunction. Increasing evidence suggests that the oral microbiome is associated with central nervous system (CNS) disorders through multiple pathways, including systemic inflammatory responses, hematogenous dissemination of periodontal pathogens and neural routes such as the trigeminal nerve [69]. Oral pathogens, particularly *Porphyromonas gingivalis*, have been detected in brain tissues and are implicated in neuroinflammatory and neurodegenerative processes, including Alzheimer's disease [70,71]. In addition, oral microbiota dysbiosis may contribute to CNS dysfunction through modulation of microglial activation and blood‒brain barrier integrity via systemic immune signaling [72]. However, whether these oral microbiota-derived signals can reach and directly modulate central gustatory processing regions remains unproven, and no experimental evidence has yet confirmed an effect on gustatory centers in the brain. Therefore, this pathway should currently be considered a putative mechanism that requires further validation through dedicated mechanistic studies.

Additionally, the maintenance of oral microenvironmental homeostasis involves the neuro-salivary regulatory axis. Specifically, gustatory stimuli can precisely modulate both the flow rate and compositional profile of saliva via cholinergic neural reflexes [73]. Salivary glycoproteins, such as mucins, form the acquired pellicle which provides attachment sites for certain commensal bacteria while simultaneously interfering with the colonization of specific pathogens through binding and aggregating mechanisms. Secretory immunoglobulin A (SIgA) in saliva can mediate the selective adherence of bacteria [74]. In parallel, a repertoire of antimicrobial proteins and peptides, including lactoferrin and peroxidases, is released. These components collectively constitute a multi-layered and synergistic defensive system against microorganisms [75].

## Role of taste-oral microbiome interactions in human diseases

**Figure 2.**
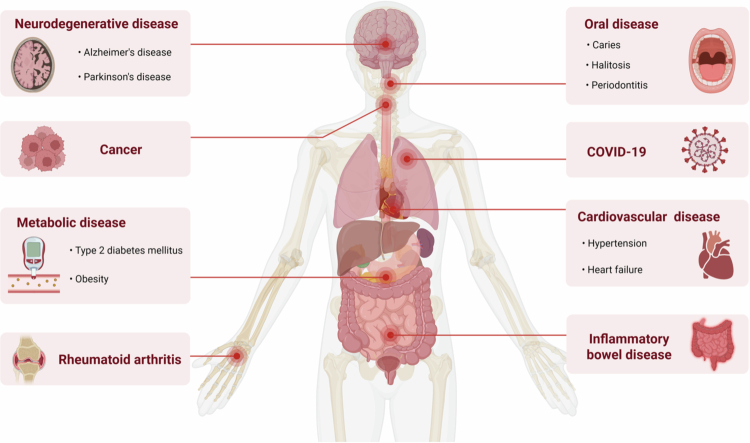
Human diseases associated with taste-oral microbiome interactions. Taste-oral microbiome interactions are linked to various diseases including oral disease, extraoral inflammatory diseases, cardiovascular disease, metabolic disease, cancer and neurodegenerative disease.

Oral microbiota and taste epithelium engage in multifaceted interactions through diverse mechanisms. These interactions not only modulate local taste function but also extend their influence to broader physiological systems. Evidence indicates that such cross-talk may contribute to the pathogenesis and progression of both oral diseases and systemic conditions (Table 2), thereby highlighting its potential role in human health (Figure 2).

**Table 2.** Taste-oral microbiome interactions and oral/systemic disease associations.

Disease category	Key oral microbiota	Relevant findings	Study design	Microbial sampling site	Taste assessment methods	Reference
caries	*Streptococcus mutans*	Compared with caries-free controls, children with dental caries exhibit reduced sweet taste sensitivity. This altered gustatory perception is closely associated with oral microbial dysbiosis, particularly an enrichment of *Streptococcus mutans* and other cariogenic taxa, and correlates with caries status, suggesting a functional interplay between taste perception, oral microbiota composition and caries development.	Cross-sectional case–control study	Oral rinse samples	Whole-mouth sucrose threshold & preference test	Jurczak et al. [41]
	Broad oral bacterial and fungal taxa such as *Capnocytophaga* and *Streptococcus*	Variants in genes that encode components of the GPCR signaling cascade may interfere with the host-microbial interactions mediated by taste receptors, as well as with taste preferences and dietary choices, leading to increased risk of S-ECC.	Cross-sectional case–control study	Supragingival plaque	No direct taste phenotype testing was performed. Taste perception was inferred indirectly via genetic variants in taste signaling pathway genes.	de Jesus et al. [76]
	Bacteria such as *Streptococcus mutans* and *Neisseria*; Fungi including *Candida albicans* and *Candida dubliniensis*	S-ECC risk and composition of the plaque microbiome can be partially influenced by genetic variants in genes related to taste sensation.	Cross-sectional case–control study	Supragingival plaque	No direct taste phenotype testing was performed. Taste perception was inferred indirectly via genetic variants in taste-related genes.	de Jesus et al. [77]
halitosis	*Prevotella, Tannerella, Filifactor and Mycoplasma*	*hTAS2R38* polymorphisms contribute to the development and treatment outcome of halitosis via modulating oral microbiota.	Cross-sectional study, prospective cohort study and in vitro experiments	Saliva	No direct taste perception testing was performed. hTAS2R38 polymorphisms were used as a genetic proxy for bitter taste phenotypes.	Mei et al. [78]
Rheumatoid Arthritis (RA)	Diverse oral microbiota including *Porphyromonas* and *Aggregatibacter,*	TAS2R38 polymorphisms are associated with distinct alterations in buccal microbiome composition in rheumatoid arthritis.	Cross-sectional case‒control study	Buccal mucosa	No direct taste perception testing was performed. hTAS2R38 polymorphisms were used as a genetic proxy for bitter taste phenotypes.	de Jesus et al. [79]
obesity	Lactobacillaceae, TM7 and Bacteroidaceae families	The CVP microbiota in NT subjects exhibits a higher capacity for inducing local inflammation compared to T individuals. Obesity amplified the phenotypic differences found between T and NT. Specific bacterial metabolic pathways (i.e. phosphotransferase and simple sugar transport systems) are higher in obese non-tasters.	Cross-sectional study	Circumvallate papillae	3-alternative forced-choice procedure (3-AFC) linoleic acid threshold test	Besnard et al. [47]
Type 2 diabetes	Oral microbiota including Proteobacteria, Porphyromodaceae and *Clostridium_XIV*	The diabetic patients with defective fatty taste detection are characterized by a specific microbiota metabolism at the circumvallate papillae levels.	Cross-sectional study	Circumvallate papillae	3-alternative forced-choice procedure (3-AFC) linoleic acid threshold test	Besnard et al. [80]
Cancer	*Enterococcus, Streptococcus parasanguinis, Veillonella parvula and Streptococcus mutans*	Miraculin intake could modify the oral microbiome in patients with cancer and dysgeusia, which may contribute to a better immune response.	Randomized controlled trial	Saliva	Electrogustometry and taste strip tests	Plaza-Diaz et al. [81]

### Oral diseases

The interaction between oral microbial communities and gustatory function may be implicated in the pathogenesis and clinical presentation of various oral diseases, such as dental caries, periodontal diseases and halitosis.

Dental caries is a bacterial infectious disease primarily driven by cariogenic bacteria such as *Streptococcus mutans*. Recent evidence indicates that taste gene polymorphisms and sweet taste sensitivity are associated with susceptibility to caries, particularly early childhood caries (ECC). In a genetic association study investigating severe early childhood caries (S-ECC), de Jesus and colleagues reported that polymorphisms in taste-related genes were significantly associated with S-ECC susceptibility and changes in plaque microbiome composition [77]. The research team further identified an association between mutations in genes encoding downstream taste signaling transduction components (including *GNAQ*, *GNAS*, *GNAT3*, *PLCB2*, *RALB* and *RAC1*) and the composition of oral fungal and bacterial communities [76]. Supporting this, a cross-sectional study in 63 preschool children indicates that preschool children with higher sucrose taste thresholds are over ten times more likely to be infected with *Streptococcus mutans* and nearly nine times more likely to develop dental caries. Additionally, children with reduced sensitivity to sucrose tend to consume sweets more frequently [41].

Periodontitis is a prevalent oral condition among humans, primarily driven by a characterized polymicrobial dental-plaque community that promotes destructive host responses through synergy and dysbiosis [82,83]. In a multicenter cross-sectional clinical study involving adult patients, Cassiano et al. observed that individuals with self-reported periodontitis exhibited impaired taste perception compared with periodontally healthy controls [84]. Furthermore, a prospective clinical study indicates that individuals with periodontitis experience a higher prevalence of taste impairment than gingivitis and gingivally healthy groups, and non-surgical periodontal treatment can significantly improve their taste perception [85]. On the other hand, taste-related pathways also exert influence on oral microbiota, thereby affecting onset and progression of periodontitis. Gingival solitary chemosensory cells (gSCCs), as immune sentinels of the oral cavity, express taste receptors and detect microbial signals, thereby initiating local immunoinflammatory responses that contribute to the alleviation of periodontitis progression [86]. It has been corroborated that defects in the taste transduction pathway (Gnat3^–/–^) or deficiency of gSCCs can induce oral microbiota imbalance and intensified periodontal damage in mice, whereas stimulation of bitter taste receptors in gSCCs enhances antimicrobial peptide production and ameliorates periodontitis [60]. Similarly, there is also research showing that the activation of Tas2r143-mediated bitter taste signaling in gingival fibroblasts presenting SCC activity can alleviate periodontitis in mouse [86]. In human gingival fibroblasts, TAS2R16 activation can suppress LPS-induced cytokine expression by downregulating intracellular cAMP and antagonizing NF-κB signaling cascade, which may protect periodontal tissue injury [87].

Additionally, halitosis, a disease characterized by an unpleasant odor emanating from the oral cavity, is also thought to be associated with oral microorganisms and taste perception. This condition is believed to arise from intricate interactions between microbes and substrates, as well as between different microbial species within the oral environment [88,89]. Recent research has indicated that bitter taste receptors play a role in the pathogenesis of halitosis. Bitter taste receptor TAS2R38, encoded by gene *hTAS2R38*, determines the reactivity to 6-*n*-propylthiouracil (PROP). Common haplotypes PAV and AVI give rise to three taste sensitivity genotypes: PAV/PAV (supertasters), PAV/AVI (intermediate tasters) and AVI/AVI (nontasters) [90]. In a combined cross-sectional and 6-month prospective cohort study, Mei et al. demonstrated that *hTAS2R38* polymorphisms contribute to the development and treatment outcomes of halitosis by modulating the oral microbiota, notably by promoting the colonization and persistence of bacteria such as *Prevotella* and *Tannerella* in AVI/AVI individuals [78]. Furthermore, some studies have reported a correlation between halitosis and taste disorders [91,92], but the overall strength of the evidence is insufficient [93].

### Extraoral inflammatory diseases

Inflammatory diseases are broadly defined as conditions characterized by a dysregulated or abnormal inflammatory response that leads to tissue damage, organ dysfunction and clinical symptoms [94]. Recent studies have identified associations among microorganisms, taste and certain inflammatory diseases including autoimmune inflammatory diseases such as Sjögren's syndrome and rheumatoid arthritis [79], as well as autoinflammatory diseases such as inflammatory bowel disease [95,96]. Supporting this link, for example, research using the MRL/lpr mouse model of autoimmune disease have revealed defects in peripheral taste structure and function, with type II taste receptor cells being selectively impacted [97]. Furthermore, clinical observations have shown alterations in the oral microbiota of patients with systemic autoimmune diseases, suggesting the potential relation between oral dysbiosis and disease progression [98].

Previous studies have established the link between the autoimmune disease rheumatoid arthritis (RA) and the oral microbiome. Specifically, *P. gingivalis* produces a unique citrullinating enzyme that contributes to the generation of autoantibodies involved in the pathogenesis of RA [2]. Research has demonstrated that patients with RA exhibit a higher subgingival bacterial load, increased microbial diversity and a greater abundance of pathogenic species compared to healthy controls. Moreover, in a clinical case–control study of RA patients, researchers reported an increased abundance of pathogenic oral bacteria including *Fusobacterium nucleatum* and *Treponema socranskii*, and microbial dysbiosis was found to be correlated with disease severity indicators such as the number of tender and swollen joints [99]. In light of the immune function associated with bitter taste receptors, de Jesus et al. explored the interrelationship between oral microorganisms, bitter taste receptors and RA. Their research identified a notable overrepresentation of the functional PAV/PAV genotype in individuals with RA compared to population controls reported in the literature. Furthermore, the buccal bacteriome composition in RA patients, which differs from that of non-RA controls (e.g. the relative abundance of *Porphyromonas*), was also observed to vary according to the *TAS2R38* genotype [79]. These findings imply that oral dysbiosis and mucosal innate immunity involving TAS2Rs may play a role in RA pathogenesis, suggesting that *TAS2R38* polymorphisms could affect the oral microbial composition in RA patients.

In addition, patients with Sjögren's syndrome (SS), a chronic autoimmune disorder [100], may exhibit various oral manifestations, including dysgeusia and Candida overgrowth [101]. A recent study has indicated that individuals with Sjögren's syndrome frequently experience dysregulation of the oral microbiota, with *Veillonella parvula* emerging as a potential biomarker for the condition [102]. Nevertheless, there is currently no definitive evidence establishing a causal link between the taste disorders observed in patients with Sjögren's syndrome and alterations in the oral microbiota. This might be caused by the interaction of multiple factors such as salivary hypofunction and involvement of the nervous system [100].

The oral cavity and the intestine, both integral components of the digestive system, are closely interconnected. Inflammatory bowel disease (IBD) is a chronic inflammatory disease of the gastrointestinal tract. Melis et al. identified that patients with IBD exhibit impaired perception of sweet, salty, bitter, umami and fatty tastes, alongside an enhanced perception of sour taste, potentially linked to disruptions in the salivary protein gustin CAVI. Further investigations suggest that zinc deficiency, in conjunction with alterations in gustin CAVI, may contribute to oral dysbiosis in IBD [96]. Previous research has demonstrated that the ectopic colonization of oral bacteria in mouse gut can provoke significant immune responses and intestinal inflammation [103,104]. However, recent studies propose that the relationship between translocated oral bacteria in the gut and disease pathogenesis may be mediated by the depletion of gut commensals [105].

COVID-19 has been increasingly recognized as a multisystem infectious disease with prominent inflammatory characteristics, involving both local mucosal immunity and systemic immune dysregulation. COVID-19 has been widely reported to cause gustatory dysfunction, particularly dysgeusia and hypogeusia, which may persist beyond the acute phase of infection [33]. A systematic review and meta-analysis including 235 studies and 138,015 COVID-19-positive patients estimated that the prevalence of taste dysfunction was approximately 36.62% [106]. Emerging evidence suggests that SARS-CoV-2 infection can affect taste perception through multiple mechanisms, including inflammation-mediated injury of taste receptor cells, epithelial dysfunction and systemic immune activation [107,108]. Huang et al. integrated oral single-cell datasets, tissue validation and saliva analyzes, demonstrating that SARS-CoV-2 can involve oral epithelial and salivary gland tissues, with salivary viral load being associated with taste loss [109]. In parallel, COVID-19 has been associated with significant alterations in the oral microbiome, including altered microbial diversity and composition [110,111]. Iebba et al. reported reduced oral microbial richness, altered *β*-diversity and inflammation-related changes in the oral microbiota of patients with COVID-19, with *Prevotella salivae* and *Veillonella infantium* identified as distinctive in COVID-19 patients [112]. These changes may further contribute to dysregulated taste perception and oral homeostasis. Importantly, persistent taste disturbances may also influence dietary behavior, thereby indirectly reshaping the oral microbial community. However, direct causal evidence linking SARS-CoV-2-induced oral microbiome alterations to dysgeusia remains insufficient. Therefore, COVID-19-associated dysgeusia should currently be regarded as an emerging and clinically relevant phenomenon that warrants further longitudinal and mechanistic investigation.

### Cardiometabolic diseases

Cardiometabolic diseases (CMDs) comprise a group of interrelated disorders, encompassing metabolic conditions such as obesity and type 2 diabetes mellitus (T2DM), alongside cardiovascular complications like heart failure (HF) [113]. Extensive research has linked various taste sensations such as fat taste to CMDs [114]. For instance, obesity correlates with reduced orosensory detection of long-chain fatty acids (LCFAs) [115], while oleanolic acid exerts anti-obesity and metabolic protective effects partly by regulating CD36 mRNA expression in taste bud cells and modifying gustatory lipid perception [116]. Individual taste perception differences influence habitual food consumption [10], with massive studies confirming a link between sweet taste perception and sweet food intake [117,118]. The reduction of peripheral sweet signals leads to compensatory behavior, wherein individuals seek stronger stimulation [118] that drives increased intake of high-calorie and sugar-rich foods, elevating risks of overweight, obesity and metabolic complications [10,35,118]. Altered salt taste sensitivity similarly contributes to hypertension and cardiovascular diseases via excessive sodium intake [119]. Furthermore, it has been demonstrated that high-cholesterol diet can upregulate CD36 scavenger receptors in rabbit aortic tissue which linked to atherosclerosis and foam cell formation [120], and the intake of polyunsaturated and saturated fatty acids has been independently linked to one-year all-cause mortality chronic heart failure patients [121].

Similar to the well-established role of diet in shaping the gut microbiota [122], there is also a proven connection between habitual diet and the oral microbiota. Studies have indicated that oral microbiome profiles are associated with sugar intake and taste preference genes, such as *TAS1R1* and *GNAT3*[52]. Cattaneo et al. analyzed the relationship between taste perception, food intake and oral microbiome composition in adults, finding that energy and macronutrient intake were significantly associated with several bacterial groups. Oral microbiota may influence dietary intake and systemic health by affecting taste perception, with host–microbe interactions in olfactory perception and appetite potentially contributing to this process [43,123].

**Figure 3.**
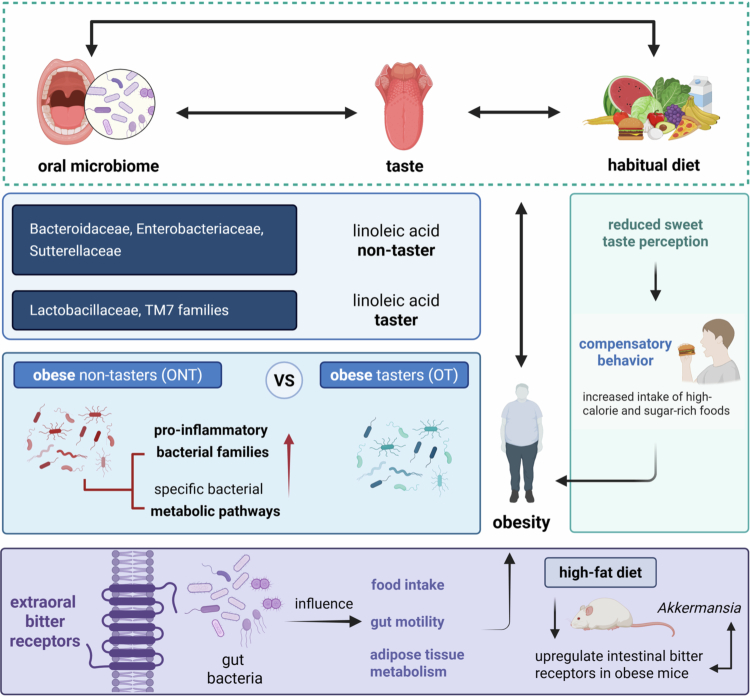
Associations among oral microbiome, taste and obesity. Individuals with varying sensitivities to linoleic acid exhibit distinct microbial profiles surrounding circumvallate papillae, with specific bacterial metabolic pathways differing between obese tasters and obese non-tasters. Taste-related dietary changes may increase obesity risk. Extraoral bitter taste receptors interact with gut microbiota to influence obesity through food intake, gut motility and adipose tissue metabolism. Animal studies have shown that a high-fat diet upregulates intestinal bitter taste receptors in obese mice, and their expression is associated with the gut microbiome, including a positive correlation with *Akkermansia*.

Extensive research has established connections between taste perception and oral microorganisms in the context of CMDs such as obesity (Figure 3). Individuals with obesity who possess specific gustatory papillae microbiota exhibit a diminished capacity to detect lipids. An observational study focusing on human fatty taste sensitivity identified that the Lactobacillaceae and TM7 families were predominant in the circumvallate papillae microbiota of the taster (T) group. In contrast, non-taster (NT) individuals were characterized by an increased presence of the Bacteroidaceae family and greater bacterial diversity, including members of the Enterobacteriaceae and Sutterellaceae families. Obesity exacerbated the phenotypic differences observed between T and NT groups. A shift in host-associated microbiota towards pro-inflammatory bacterial families, such as Bacteroidaceae, Sphingomonadaceae and Prevotellaceae, was noted in obese non-tasters (ONT) compared to obese tasters (OT) [47]. A cross-sectional study conducted by Besnard and colleagues has demonstrated that diabetic patients with impaired fatty taste detection exhibit specific microbiota metabolism at the level of the circumvallate papillae. A significant positive correlation with linoleic acid detection threshold was observed for the phylum Proteobacteria and genus *Clostridium_XIV*, whereas a negative correlation was found for the family Porphyromodaceae. This suggests that the composition of the circumvallate papillae microbiota may influence fatty taste sensitivity of diabetic patients [80].

Recent studies have further elucidated the role of extraoral taste receptors in CMDs, highlighting their interactions with microorganisms [124]. Extraoral bitter receptors have been shown to interact with gut bacteria, thereby influencing food intake and gut motility, both of which are factors in obesity risk [125]. In obese mice, a high-fat diet has been found to upregulate the expression of Tas2r116 and Tas2r138 in the large intestine, with this expression being positively correlated with the presence of *Akkermansia*, a Gram-negative mucus-resident bacterium [126,127]. Numerous studies have demonstrated the positive effects of bitter agonist polyphenols on type 2 diabetes and obesity. The intestinal microbiota can modulate the interaction between TAS2Rs and bitter agonists such as polyphenols, significantly impacting adipose tissue metabolism [128]. These findings support the hypothesis that intestinal TAS2Rs maintain luminal homeostasis by detecting bacterial presence, potentially alleviating metabolic complications through this protective mechanism.

### Cancer

Emerging evidence indicates that the intricate interaction between oral microbiota and taste system holds substantial implications for the initiation, treatment and prevention of cancer, which may offer a novel perspective in oncological research.

Oral microbiota may influence the onset and progression of cancer through pathways associated with taste bud renewal including the Hedgehog (Hh) signaling pathway. Shh is expressed in taste bud cells as one of the Hh ligands [129], and the Hh pathway is believed to play a regulatory role in the renewal of taste organs and the maintenance of taste sensation [130]. Dysregulated Hh signaling is implicated in numerous cancers [131]. Jia et al. discovered that the cultured media of the periodontal pathogen *P. gingivalis* can induce the malignant transformation of normal esophageal epithelium via the Sonic hedgehog (Shh) pathway [132].

Moreover, systemic cancer treatments, including chemotherapy, immunotherapy and radiotherapy, are associated with oral microbiome and adverse side effects that can impact gustatory function. For instance, while Hh pathway inhibitors demonstrate efficacy as an innovative therapeutic strategy, they have also been linked to adverse effects, including taste disorders [129]. Research indicates that cancer patients undergoing treatment who experience dysgeusia exhibit oral dysbiosis characterized by reduced bacterial diversity and richness, with a predominance of *Streptococcus pneumoniae*, *Streptococcus thermophilus* and *Veillonella parvula* [81]. A novel intervention has been suggested to ameliorate taste disorders in cancer patients. Studies have demonstrated that regular intake of dried miracle berries (DMBs), which contain the taste-modifying glycoprotein miraculin, can modify the oral microbiome in patients with cancer and dysgeusia, potentially supporting an appropriate immune response [81]. Furthermore, Wang et al. demonstrated in murine models that probiotic *Streptococcus salivarius* K12 (SsK12) alleviated radiation-induced oral mucositis and taste dysfunction by modulating the oral microbiota [133]. Clinical trials have further validated the efficacy of SsK12, demonstrating its ability to inhibit opportunistic pathogens while enhancing oral commensals during radiotherapy [134].

In addition, brassica vegetables with bitter taste have been identified to provide various health benefits including cancer prevention. Isothiocyanates (ITCs), as compounds responsible for the characteristic bitterness in brassica vegetables, have been observed to inhibit the initiation and delay the progression of cancer across its key developmental stages [135]. Individuals with heightened sensitivity to bitter tastes may avoid consuming vegetables rich in anti-tumor and anti-oxidant compounds, potentially increasing their susceptibility to cancer [136]. Meanwhile, individuals with varying sensitivity to bitter taste have been demonstrated to exhibit differences in oral microbiota composition. A study examining the composition of the tongue microbiota in individuals with varying PROP reactivity found that in the super-tasters group (those with high PROP reactivity), five oral bacterial genera are significantly overrepresented including the Gram-positive genera *Actinomyces*, *Oribacterium*, *Solobacterium* and *Catonella* and the Gram-negative *Campylobacter* [40]. Recent research indicates that alterations in the microbiome induced by brassica-rich diets may enhance the delivery of health benefits including cancer prevention [135].

### Neurodegenerative diseases

Emerging evidence points to an association between taste perception and neurodegenerative diseases. In a clinical observational study, Parkinson's disease patients show a higher prevalence of PROP non-taster status linked to the *TAS2R38* locus [137], while Alzheimer's disease is often associated with a progressive taste disorder [138]. Research has identified human bitter taste receptors within the central nervous system [139], suggesting its potential roles in brain homeostasis and neuroinflammation modulation [140]. Neuroprotective bitter compounds like resveratrol exert anti-inflammatory effects partly through TAS2Rs [141], whereas the downregulation of taste signaling components may contribute to neuroinflammation by elevating oxidative stress and NF-κB activity [142].

The oral-gut-brain axis is increasingly recognized as a significant pathway in the pathogenesis of neurodegenerative diseases [143–145]. There is a direct and multifaceted interaction between oral and intestinal microbiota [18]. Meanwhile, it has been demonstrated that the gut microbiota composition varies across different *TAS2R38* genotypes in PD patients [146]. Specifically, compared to the PAV/PAV genotype, the AVI/AVI genotype is related to a decrease in bacterial alpha-diversity with a significant reduction in the *Clostridium* genus. Nevertheless, there is currently no direct evidence to suggest that peripheral interactions between oral microbiota and taste perception contribute to disease development. No significant difference was observed in the expression of taste markers in taste bud cells between wild-type mice and the *App* knock-in mouse models of Alzheimer's disease [147]. Numerous studies propose that taste dysfunction may manifest predominantly in later disease stages with cortical involvement rather than drive early pathogenesis [68,148,149].

## Conclusions and perspectives

The oral microbiome-taste interaction in human diseases is an area of research that is rapidly advancing. Oral microorganisms can regulate taste perception mainly through microbial metabolism, immunoinflammatory mediation, alteration of taste receptors expression and the formation of physical barriers. Furthermore, changes in taste perception and taste receptors may regulate the colonization and adhesion of oral microbiota by affecting dietary behavior and local immune microenvironment, establishing a bidirectional regulatory loop. This interaction network may play an important role in both oral and systemic diseases, including caries, halitosis, inflammatory diseases, cardiometabolic diseases and cancer.

Nevertheless, current evidence remains largely associative, and causal relationships as well as underlying mechanisms linking the oral microbiome, taste perception and systemic diseases are not yet established. The bidirectional interactions between host systemic conditions and the oral microbiome further complicate causal inference. Systemic diseases, including diabetes and obesity, can influence the composition and function of the oral microbiome as well as taste perception by altering metabolic states and inflammatory levels, while certain antidiabetic and hypolipidemic medications may have adverse effects on taste acuity [80]. It should also be noted that the strength of evidence supporting the involvement of the taste-oral microbiome axis is not uniform across the disease categories discussed in this review. Based on the synthesis of available studies, relatively stronger and more consistent associations are observed in oral diseases and cardiometabolic disorders. Multiple independent studies have reported links among oral microbiome composition, taste perception and disease-related phenotypes, with some support from mechanistic and functional evidence. Evidence for extraoral inflammatory diseases is moderate and mainly derived from observational associations combined with partial mechanistic plausibility, reflecting interactions among oral dysbiosis, mucosal immunity, salivary alterations and taste dysfunction. In contrast, evidence for cancer and neurodegenerative diseases remains comparatively limited and should be interpreted as exploratory, because most available data are derived from indirect associations, treatment-related observations, or emerging mechanistic hypotheses. Therefore, the involvement of the taste-oral microbiome axis in these conditions should be interpreted with caution. Future studies are required to clarify the causal and mechanistic roles of this axis in the context of specific disease, including potential involvement of extraoral taste receptors and oral-gut microbial interactions.

The heterogeneity in research methodologies and study populations remains a major challenge when interpreting current evidence on the taste-oral microbiome interactions. In particular, the lack of standardized gustatory assessment methods and unified evaluation criteria limits the comparability of findings across studies. Existing studies have employed diverse approaches, including whole-mouth taste tests [150], electrogustometry [151,152] and validated psychophysical tools such as taste strip tests [153]. These methods assess different dimensions of gustatory function including detection thresholds, intensity ratings and hedonic preference [117]. However, no universally accepted gold standard currently exists for taste function evaluation. Among these methods, taste strip testing is increasingly considered one of the most standardized and clinically applicable approaches due to its high reproducibility and ease of cross-population implementation, whereas electrogustometry provides complementary objective threshold measurements but is limited in capturing suprathreshold taste perception and complex taste quality processing [150]. Therefore, future studies would benefit from a combined methodological framework integrating psychophysical taste testing with objective electrophysiological measurements, enabling a more comprehensive characterization of taste function. In parallel, inconsistent oral microbiome sampling strategies further complicate interpretation. Although most studies primarily collect samples from the tongue dorsum [123], other investigations utilize saliva [41], buccal mucosa [79], or supragingival plaque. These sites represent distinct ecological niches with different microbial stability and functional relevance. In particular, the tongue dorsum is widely regarded as a relatively stable microbial reservoir with site-specific colonization patterns, whereas saliva reflects a composite microbial community derived from multiple oral habitats and therefore provides lower spatial resolution and is more susceptible to sampling-related compositional bias [14,154]. Furthermore, we emphasize that oral microbiome composition is strongly influenced by host-related and environmental variables, including age, dietary patterns, medication use and geographic background [155], all of which are also key determinants of taste perception. These differences should therefore be carefully considered when interpreting associations between oral microbial composition and taste-related phenotypes. Future studies should therefore adopt multivariate statistical frameworks and stratified analyzes to better control for confounding and improve statistical validity in microbiome-taste association studies.

Addressing these limitations will be essential for future clinical translation. From a clinical and therapeutic perspective, the taste-oral microbiome axis may provide new opportunities for disease risk assessment and individualized intervention. Integrated evaluation of gustatory function and oral microbiome profiles may help identify individuals who are susceptible to oral or specific systemic disorders and monitor treatment-related taste dysfunction. In addition, strategies aimed at restoring a balanced oral ecosystem, such as optimized oral hygiene, periodontal therapy, probiotics, postbiotics and other microbiome-modulating approaches, may have potential to improve taste function and oral or systemic health outcomes. Taste-guided nutritional interventions may also be useful, particularly for individuals with altered sweet, salt, bitter, or fat taste sensitivity, as these changes may influence dietary behavior and metabolic risk. Moreover, in patients with cancer or chronic systemic diseases, modulation of the oral microbiome may represent a supportive strategy to alleviate dysgeusia, improve food intake and enhance quality of life. Nevertheless, these clinical applications remain largely exploratory, and future longitudinal studies and controlled clinical trials are required to determine the efficacy, safety and mechanistic basis of interventions targeting the taste-oral microbiome axis.

In conclusion, the oral microbiome is not only crucial for maintaining oral health but also serves as a significant modulator of taste perception and overall human health. By elucidating the complex interactions between oral microbiome and taste perception, we can gain deeper insights into the role that they play in the onset and development of certain diseases, thus paving the way for novel microbiome-targeted therapeutic strategies to improve taste disorders and associated health conditions.
